# *Bcmimp*1, a *Botrytis cinerea* Gene Transiently Expressed *in planta*, Encodes a Mitochondrial Protein

**DOI:** 10.3389/fmicb.2016.00213

**Published:** 2016-02-26

**Authors:** David Benito-Pescador, Daniela Santander, M. Arranz, José M. Díaz-Mínguez, Arturo P. Eslava, Jan A. L. van Kan, Ernesto P. Benito

**Affiliations:** ^1^Instituto Hispano-Luso de Investigaciones Agrarias – Departamento de Microbiología y Genética, Universidad de SalamancaSalamanca, Spain; ^2^Facultad de Ciencias Agropecuarias y Ambientale, Universidad Técnica del NorteIbarra, Ecuador; ^3^Departamento de Microbiología y Genética, Universidad de SalamancaSalamanca, Spain; ^4^Laboratory of Phytopathology, Wageningen UniversityWageningen, Netherlands

**Keywords:** *Botrytis cinerea*, plant-pathogen interaction, mitochondria, ROS, pathogenicity

## Abstract

*Botrytis cinerea* is a widespread necrotrophic fungus which infects more than 200 plant species. In an attempt to characterize the physiological status of the fungus *in planta* and to identify genetic factors contributing to its ability to infect the host cells, a differential gene expression analysis during the interaction *B. cinerea*-tomato was carried out. Gene *Bcmimp*1 codes for a mRNA detected by differential display in the course of this analysis. During the interaction with the host, it shows a transient expression pattern with maximal expression levels during the colonization and maceration of the infected tissues. Bioinformatic analysis suggested that BCMIMP1 is an integral membrane protein located in the mitochondrial inner membrane. Co-localization experiments with a BCMIMP1-GFP fusion protein confirmed that the protein is targeted to the mitochondria. *ΔBcmimp*1 mutants do not show obvious phenotypic differences during saprophytic growth and their infection ability was unaltered as compared to the wild-type. Interestingly, the mutants produced increased levels of reactive oxygen species, likely as a consequence of disturbed mitochondrial function. Although *Bcmimp*1 expression is enhanced *in planta* it cannot be considered a pathogenicity factor.

## Introduction

*Botrytis cinerea* is the causal agent of gray mold disease in a broad range of dicotyledonous plants (Williamson et al., 2007). It colonizes senescent or wounded tissues but is also able to infect healthy plants, causing serious damage in fruits and vegetables in open fields and in greenhouses, both during pre- and post-harvest (Droby and Lichter, 2007). *B. cinerea* is considered to be an exemplary necrotroph, as it meets all the classic criteria of this type of pathogen (van Kan, 2006; Tudzynski and Kokkelink, 2009). Its infection strategy includes killing of host cells by means of the secretion of cell wall degrading enzymes and toxic metabolites that induce cell death in advance of the invading hyphae (Clark and Lorbeer, 1976; Govrin and Levine, 2000; Kars and van Kan, 2007). The fungus is then able to derive nutrients from the dead tissues. Necrotrophs are seen as pathogens less adapted to the host than biotrophs, widely accepted to establish complex and sophisticated interactions with the host in the course of which both organisms interchange signals that can modulate the responses and behavior of each other. This active communication leads to adaptation and to co-evolution of host and pathogen. However, evidence accumulated during the last decade has illustrated that the interactions between necrotrophs and their hosts are more complex and subtle than initially thought. The analysis of the interactions with *Arabidopsis* and tomato indicates that *B. cinerea* triggers the plant programmed cell death and exploits this response for its own benefit (Govrin and Levine, 2000; Hoeberichts et al., 2003; van Baarlen et al., 2007). Remarkably, it has been demonstrated that the related necrotroph *Sclerotinia sclerotiorum* is able to modulate, via oxalic acid, the plant defense responses by first suppressing and later inducing host reactive oxygen species (ROS) production, facilitating the establishment and progress of the pathogen on the plant tissues (Kim et al., 2008; Williams et al., 2011).

*B. cinerea* has become a model system to investigate the nature of the pathogenicity determinants of necrotrophs. Due to its economic importance, the biology, epidemiology and chemical control of the fungus have been studied extensively (Coley-Smith et al., 1980; Elad et al., 2007). During the last two decades much cytological, biochemical and molecular research has been performed, significantly improving our understanding of the different stages of the infection process. In order to identify factors important for pathogenicity, two general strategies have been considered. The classical “candidate gene” approach proposes the analysis of genes whose participation in the infection process might be assumed based on previous research. In the case of *B. cinerea* a large number of genes involved in cell wall degradation, synthesis of phytotoxic compounds, signaling, production, and detoxification of ROS have been investigated as candidate virulence factors (for a review, see Tudzynski and Kokkelink, 2009). A second, “non-biased” approach is based on functional criteria. Two experimental strategies have been considered to obtain information about novel functions related to pathogenicity. First, the isolation of mutants altered in pathogenicity, obtained either by chemical treatment (Weeds et al., 1999) or by random insertional mutagenesis (Giesbert et al., 2012). And second, the identification and analysis of genes differentially expressed *in planta*, a strategy based on the assumption that the differential expression of a gene during the interaction of a fungus with its host, is an indication of that gene playing a role in the infection process. Several techniques have been applied in *B. cinerea*: differential display RT-PCR (Benito et al., 1996), suppression subtractive hybridization (Gronover et al., 2004), macroarray hybridization (Viaud et al., 2003; Schumacher et al., 2008) and more recently, microarray hybridizations (Amselem et al., 2011) and genome-wide transcriptomic analysis by RNA Seq (Kong et al., 2015; Kelloniemi et al., 2015). In the work by Benito et al. (1996) a comparative analysis of the expression pattern of *B. cinerea* cultured *in vitro* with its expression pattern during the interaction with tomato was carried and several cDNA fragments were detected that are derived from *B. cinerea* genes whose expression is enhanced *in planta*. One of those cDNA fragments, named ddB47, detected on a northern blot analysis two different transcripts, both displaying transient expression patterns during the interaction.

Mitochondria are double-membrane-bound organelles that participate in numerous and important cellular processes. They are highly organized organelles containing proteins encoded either by the nuclear or the mitochondrial genome. These proteins are distributed among four mitochondrial subcompartments: outer membrane (OM), inner membrane (IM), matrix and intermembrane space (IS). The proteins and protein complexes within each subcompartment are involved in specific functions. For example, enzymes involved in the TCA cycle and respiratory chain are located within the mitochondrial matrix and IM. Many regulatory proteins required for mitochondrial movement and apoptosis are found in the mitochondrial OM. The IS represents the smallest subcompartment, however, it plays important roles in transport processes, in the assembly of the respiratory complexes and in coordinating key steps in programmed cell death. Proteomic analysis of purified mitochondria has identified about 900 (in yeast) and 1100 (in mouse) different proteins (Reinders et al., 2006; Pagliarini et al., 2008). These values probably represent underestimates and it is accepted that the yeast and animal mitochondria host approximately 1000 and 1500 distinct proteins, respectively (Meisinger et al., 2008). Functional information has been gained for many of them. However, the function of a large number of mitochondrial proteins remains unknown (Reinders et al., 2006; Pagliarini et al., 2008).

In this work we describe the isolation and functional characterization of *Bcmimp*1, identified in the course of a differential gene expression analysis during the interaction of *B. cinerea* with tomato (Benito et al., 1996). We show that *Bcmimp*1 has orthologs only in certain taxa of fungi and that it encodes a structural mitocondrial protein which can be considered a representative member of a family of proteins previously uncharacterized.

## Materials and Methods

### Organisms and Growth Conditions

*B. cinerea* strain B05.10 is the reference laboratory strain used in the experiments described in this work. Its genome sequence is available (Amselem et al., 2011). It was grown on PDA plates containing 25% w/v of tomato leaves extracts in order to stimulate sporulation. For expression studies, the fungus was cultured in liquid Gamborg’s B5 salts medium (AppliChem, Darmstadt, Germany) supplemented with 10 mM sucrose and 10 mM KH_2_PO_4_ (pH 6.0) (B5S/SP medium). Cultures were established by inoculating flasks of liquid medium with 5 × 10^5^ spores/ml and then incubated on an orbital shaker at 22°C and 180 rpm for up to 20 h.

*Escherichia coli* strain LE392 was used to propagate the *B. cinerea* genomic DNA phage library. *E. coli* strain DH5α was used in all cloning and subcloning experiments. They were grown under previously described conditions (Sambrook et al., 1989).

Tomato plants (*Solanum lycopersicum*) cv Rome were grown in vermiculite substrate for 7 weeks in the greenhouse under a 16 h photoperiod. Bean plants (*Phaseolus vulgaris*) cv Blanca Riñon were grown in natural substrate for 2 weeks in the same conditions.

### Inoculation Experiments

For *in planta* expression studies 7 weeks-old tomato plants were spray-inoculated with a suspension of 10^6^ spores/ml in B5S/SP medium as previously described (Benito et al., 1996). At the indicated time points, leaves were sampled, frozen in liquid nitrogen and stored at -80°C until used for RNA extraction.

### Growth and Pathogenicity Tests

Growth tests of *B. cinerea* strains were carried out on B5S/SP plates. Suspensions of 10^5^ spores/ml of the different *B. cinerea* strains were prepared in B5S/SP liquid medium and 5 μl drops were deposited on the center of at least three plates per strain and experiment. Plates were incubated at 22°C in darkness and the colony diameter was measured 4 days after inoculation.

Pathogenicity tests were carried out on *S. lycopersicum* and *P. vulgaris* detached leaves. Routinely, the same spore suspensions in B5S/SP medium used for saprophytic growth analysis were also used for pathogenicity tests. 5 μl drops of 10^5^ spores/ml suspensions in B5S/SP medium were placed on the plant leaves which were allowed to dry for 30 min and then incubated at 22°C with a 16 h photoperiod and under high humidity conditions. Lesion diameter was scored at 72 h post inoculation (hpi). In each inoculation experiment 20 lesions per strain on each host were scored. Experiments were repeated three times.

### Standard Molecular Methods

The genomic library used in this work is a *B. cinerea* strain SAS56 genomic DNA library constructed in the phage vector lambda EMBL3. *B. cinerea* genomic DNA was extracted following the procedures previously described (Moller et al., 1992). Isolation of lambda DNA was performed with the Qiagen Lambda mini kit (Qiagen, Hilden, Germany). Plasmid DNA was isolated with the FastPlasmid kit (Eppendorf, Hamburg, Germany). Total RNA was isolated from frozen mycelia samples grown in B5S/SP medium using the Tri-reagent method (Chomczynski, 1993). Roche enzymes (Roche, Barcelona, Spain) were used for the DNA digestions. Ligations were performed with a ligase from Promega (Madison, WI, USA). Manufacturer’s recommendations were followed for all the enzymatic treatments. For Southern blot analysis genomic DNA was digested, size-separated on 0,7% agarose gel and blotted onto Hybond-N+ membranes following standard procedures (Sambrook et al., 1989). For northern blot preparation, samples of total RNA were electrophoresed under denaturing conditions and transferred to Amersham HybondN+ membranes. Blot hybridizations with α-^32^P-dATP-labeled probes generated either by PCR or by random-priming were performed as described earlier (Benito et al., 1996). For hybridizations under high stringency conditions a hybridization temperature of 65°C was used and washes were done in 0.1X SSC and 0.1% SDS at the same temperature. For moderately restrictive hybridization conditions, 58°C was used as the hybridization temperature and washes were done in 0,2X SSC and 0,1% SDS at the same temperature.

Polymerase chain reaction reactions were performed using the AmpliTaq Gold Polymerase from Applied Biosystems (Applied Biosystems, Foster City, CA, USA) for cloning and expression purposes. For diagnostic purposes and for PCR labeling the Taq Polymerase from Biotools was used (Biotools, Madrid, Spain). DNA sequencing was carried out with an ABI PRISM 377 automatic sequencer (Applied Biosystems, Foster City, CA, USA). When needed, PCR amplified DNA fragments were cloned in the pGEM-T vector (Promega, Madison, WI, USA). Cloning and subcloning strategies made use of plasmids pBluescript II SK + (Stratagene, La Jolla, CA, USA) as the backbone of derived constructs and of plasmid pOHT (Hilber et al., 1994) as the source of a hygromycin B resistance cassette.

### Construction of a *Bcmimp*1-GFP Fusion

**FIGURE 1:**
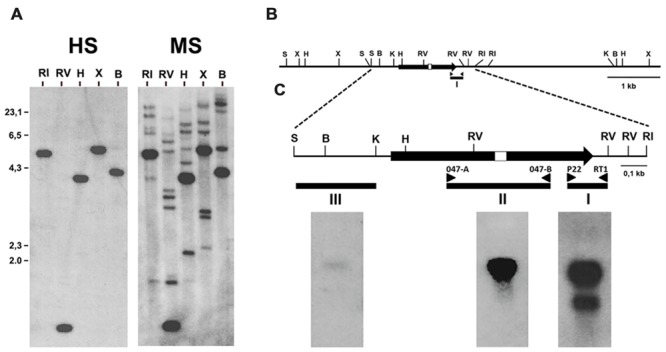
**Identification of the gene encoding the 1,4 kb mRNA detected with the cDNA fragment ddB47. (A)** Southern blot analysis of fragment ddB47. A blot containing in each lane 5 μg of *B. cinerea* genomic DNA digested with the indicated restriction enzymes was hybridized with the labeled ddB47 cDNA fragment (probe I in panels **B,C**) under high (HS) or moderate (LS) stringency hybridization conditions. **(B)** Restriction map of the *Bcmimp*1 genomic region. **(C)** Northern blot analysis of *Bcmimp*1. Three RNA blots each containing 20 μg of total RNA from *B. cinerea-*infected tomato leaves collected at 120 hpi were hybridized with probes I, II or III under high stringency conditions. B, *Bam*HI; RI, *Eco*RI; RV, *Eco*RV; H, *Hind*III; K, *Kpn*I; S, *Sal*I; X, *Xba*I.

To determine the subcellular localization of the protein encoded by gene *Bcmimp*1 a translation fusion was generated by cloning the structural region of the GFP coding gene at the 3′ end of the *Bcmimp*1 gene structural region. As a starting point plasmid p47-1, which includes a *Sal*I-*Eco*RI 1,96 kb genomic DNA fragment from *B. cinerea* containing the genomic copy of gene *Bcmimp*1 (see **Figure 1C**) cloned in pBluescript II SK+ was used. From it, an ap. 1,8 kb fragment was amplified with primers M13 Forward and OF1. Primer OF1 was designed with a sequence complementary to the sense strand of *Bcmimp*1 around the stop codon with modifications to eliminate the termination codon and to introduce a *Sal*I site (see **Table 1**). The amplified fragment was digested with *Sac*I and *Sal*I and the 1,6 kb *Sac*I-*Sal*I fragment resulting from this digestion was purified. The structural region of the GFP coding gene was obtained as a 746 nt *Sal*I-*Xba*I fragment from plasmid pMCB15 (Fernández-Ábalos et al., 1998). Both fragments were cloned together in plasmid pBluescript II SK+ digested with *Sac*I and *Xba*I, giving rise to plasmid pMAS23. The terminator region of gene *Bcmimp*1 was amplified from p47-1 with primers OF2 and M13 Reverse. The sequence of primer OF2 coincides with the sequence of the sense strand of *Bcmimp*1 and has been modified to introduce a *Xba*I site immediately upstream of the natural *Bcmimp*1 termination codon (see **Table 1**). The amplified fragment was digested with *Xba*I and *Eco*RI and the *Xba*I-*Eco*RI fragment which includes the first 295 nt of the *Bcmimp*1 termination region was purified. This fragment, together with an ap. 2,5 kb *Sac*I-*Xba*I fragment from plasmid pMAS23 containing the promoter and the structural region of gene *Bcmimp*1 fused to the structural region of the GFP gene were ligated into plasmid pBluescript II SK+ digested with *Sac*I and *Eco*RI, originating plasmid pMAS24. From it, a *Sac*I-*Sac*I fragment of 2,5 kb containing the *Bcmimp*1-GFP fusion flanked upstream by 450 nt of the *Bcmimp*1 promoter region and downstream by 220 nt of the *Bcmimp*1 terminator region was cloned into the *Sac*I site of plasmid pOHT, originating pMAS25 (**Figure 4A**), which was used to transform *B. cinerea*. That no undesired alterations had been introduced was confirmed by sequencing the whole gene fusion.

**Table 1:** Oligonucleotides used in this work.

Name	Sequence (5′-3′)
M13 Forward	TGTAAAACGACGGCCAGT
M13 Reverse	CAGGAAACAGCTATGACC
P22	GATAGCATTG
RT1	TTTTTTTTTTTGG
O47-A	AGGCCACGGATCGGTCA
O47-B	TTCTTCCTCTACCGACC
O47-Y	TTCATTGGTGCGCAGCGATC
O47-Z	TCAGTCTTGAACTGCAGGTG
OF1	GGATGCTGTCGACCTTCTTCTCATCCTTCTT
OF2	GATGAGTCTAGATAAATTAGCATCCGAGTT
Bde47-13F	GGAAGAATTGTCAGCCATGATAGA
Bde47-131R	GCCAACTTCGCCCTTCTCA
BcactA-62F	TTGCACCATCGTCGATGAAG
BcactA-131R	CCACCAATCCAGACGGAGTATT
OMAS14	TTTCGTTGGGATCTTTCG
OliC-P1	CCACTTAGTGGCACGTCGCG
HphBc	CGTCTGGACCGATGGCTGTG

*The restriction sites introduced in oligonucleotides OF1 (*Sal*I) and OF2 (*Xba*I) for directed cloning are underlined.*

#### Generation of the Gene Replacement Vector p47-13

In order to obtain deletion mutants of gene *Bcmimp*1 a gene replacement vector was generated. First, a 719 nt *Sau*3A-*EcoR*I fragment containing the last 429 nt of the structural region of gene *Bcmimp*1 and the first 290 nt of its 3′ flanking region was cloned into *BamH*I-*Eco*RI digested pBluescript II SK+. From the derived plasmid, p47-12, the insert was released upon digestion with *Xba*I and *Eco*RI. Second, a 618 nt *Sal*I-*Hind*III fragment containing 536 nt from the 5′ flanking region of gene *Bcmimp*1 and the first 82 nt of its structural region was cloned together with a 2,6 kb *Hind*III-*Xba*I fragment derived from plasmid pHOT and containing the hygromycin resistance cassette, into plasmid pBluescript II SK+ digested with *Sal*I and *Xba*I generating plasmid p47-11. From this plasmid the 3,2 kb *Sal*I-*Xba*I fragment which includes the 5′ flanking sequence of *Bcmimp*1 and the hygromycin resistance cassette was purified and then ligated together with the 737 nt *Xba*I-*Eco*RI fragment containing the 3′ flanking region of *Bcmimp*1 into plasmid pBluescritpt II SK+ digested with *Sal*I-*Eco*RI, giving rise to plasmid p47-13 (**Figure 6A**).

#### Transformation of *B. cinerea* and Selection of Transformants

Plasmids pMAS25 and p47-13 were introduced in *B. cinerea* by protoplasts transformation following the protocol described by ten Have et al. (1998) taking into consideration the modifications indicated by Reis et al. (2005). Primary transformants were individually transferred to selection plates and enriched in transformed nuclei through four successive cycles of vegetative growth on selection plates. Monosporic isolates were prepared directly from pMAS25 transformants and then analyzed by Southern blot hybridization. Transformants obtained with plasmid p47-13 were first tested by PCR for homologous recombination events at both the 5′ and 3′ flanking regions using primer combinations that would render amplification products only in the case that homologous recombination at either flanking side had occurred (oligonucleotide O47-Y and oligonucleotide OliC-P1 for recombination at the 5′ flanking region and oligonucleotide HphBc and oligonucleotide O47-Z for recombination at the 3′ flanking region) (see **Figure 6A**). Positive candidate transformants were then purified by two rounds of single-spore isolation and characterized by Southern blot analysis.

### Quantitative PCR

RNA samples were cleaned of DNA traces with the Turbo DNAfree kit (Ambion, Austin, TX, USA). cDNA was synthesized with the SuperScript III First-Strand Synthesis SuperMix for qRT-PCR (Invitrogen, Carlsbad, CA, USA) from the DNA-free RNA following manufacturer’s recommendations. Real-time PCR analysis was performed following the standard curve method. Quantitative PCR reactions were performed using the SYBR Green PCR Master Mix (Applied Biosystems, Foster City, CA, USA). For gene *Bcmimp*1, primers Bde47-13F and Bde47-131R, which amplify a 118 bp fragment from the *Bcmimp*1 coding region, were used. For gene *Bcact*A, the primers designed were BcactA-62F and BcactA-131R, which specifically amplify a 70 bp DNA fragment from the *Bcact*A coding region. The standard curve was performed with *B. cinerea* genomic DNA diluted at 100, 10, 1, 0.1, 0.01, 0.001, and 0.0001 ng. Three repetitions were made for each sample and for the standard curve. Reactions were carried out in an ABI Prism 7000 thermocycler (Applied Biosystems, Foster City, CA, USA) with the following cycling conditions: a first step at 50°C for 2 min, a second step at 95°C for 10 min to activate the AmpliTaq Gold Polymerase, and 40 amplification cycles consisting of 15 s at 95°C and 1 min at 60°C. Expression of *Bcmimp*1 was normalized against the expression of the *Bcact*A gene.

### Quantification of ROS

Levels of total ROS produced by *B. cinerea* strains were measured using the oxidant sensitive probe 2′,7′-dichlorodihydrofluorescein diacetate (H_2_DCFDA) (Molecular Probes Europe, Leiden, the Netherlands). 5 × 10^5^ spores/ml were cultured in 100 ml volumes of liquid B5S/SP medium at 22°C and 180 rpm in darkness during 5 h. Cells from 3 ml were collected by centrifugation, washed and resuspended in 600 μl of 10 mM potassium phosphate buffer (pH 7.0) containing 0,01 mM H_2_DCFDA. After incubation at 22°C for 15 min in darkness, fluorescence was measured in 200 μl aliquots using a Spectra Fluor (Tecan) spectrofluorometer at λ_EX_ = 485 nm and λ_EM_ = 535 nm. Fluorescence was recorded as absolute fluorescence units, normalized against dry weight of fungal biomass and expressed as arbitrary Fluorescence Units (FUs) per mg of dry weight (mg dw).

### Fluorescence Microscopy

For microscopy analysis, a Zeiss LSM 510 Confocal Laser Microscope was used. Conidia of the selected *B. cinerea* transformants were cultured in B5S/SP liquid medium at 22°C and 180 rpm during 5 or 16 h. Mitochondria were stained with the mitochondria-specific dye Mitotracker Red CMXRos (Invitrogen, Carlsbad, CA, USA) at a final concentration of 200 nM during 20 min at 22°C. Images were captured using the red channel. For detection of GFP fluorescence the green channel was selected.

### Bioinformatic and Statistical Analysis

Sequences were handled using Vector NTI 10 suite (Invitrogen, Carlsbad, CA, USA) and Geneious (Biomatters, Auckland, New Zealand). BLAST searches and sequence aligments were also done with Geneious. For amino acid similarity analysis a Blosum62 score matrix was considered. PSORT (Nakai and Horton, 1999) and MitoFates (Fukasawa et al., 2015) were used to predict subcellular localization and the presence of mitochondrial presequence and of MPP cleavage sites. The data generated during saprophytic and *in planta* growth were analyzed by applying an ANOVA test with both MSD and Tukey HSD posthoc analysis with the SPSS statistics 15.0 software.

## Results

### Expression Analysis, Cloning, and Sequence Characterization of *Bcmimp*1

By applying a differential display based strategy comparing the expression pattern of *B. cinerea* cultured in liquid B5S/SP medium during 16 h and its expression pattern during its interaction with tomato at different stages of infection, several cDNA fragments derived from *B. cinerea* genes differentially expressed *in planta* were identified (Benito et al., 1996). Expression analysis on a time course northern blot hybridization experiment performed on a filter prepared using total RNA samples from tomato infected leaves collected at 16, 32, 48, 72, 96, and 120 hpi revealed an intriguing observation as two mRNAs of different sizes, 1,4 and 1,0 kb, respectively, both showing enhanced expression *in planta* and displaying transient accumulation patterns, were detected with a single cDNA probe, ddB47. This cDNA fragment, ddB47, is 222 nt long and is characterized by a high -AG- content, particularly in the region corresponding to the first 145 nt, where it reaches 88% and is enriched in GAA and GAG triplets (Benito et al., 1996).

In order to determine the organization of the gene or genes encoding these two mRNAs, a Southern blot analysis was carried out. To this end a membrane to which the genomic DNA fragments derived from several restrictions had been transferred was hybridized with the labeled ddB47 cDNA fragment. Under highly restrictive hybridization conditions a single band was detected in each lane. Under moderate restrictive hybridization conditions several additional faint bands were detected (**Figure 1A**). Therefore, the mRNA from which fragment ddB47 is derived is likely encoded by a single copy gene, but there are several sequences in the *B. cinerea* genome showing some similarity to the ddB47 sequence. Hybridization under highly restrictive conditions on a genomic λ phage library yielded two partially overlapping positive phages. From these phages, a 1.960 nt *Sal*I-*Eco*RI fragment was cloned which included the 750 nt *Eco*RV-*Eco*RV fragment detected in the Southern blot hybridization (see **Figure 1B**) and its sequence was determined. Within this fragment the sequence of the cDNA fragment ddB47 was identified.

Sequence analysis indicated that only one of the six frames could be transcribed into an mRNA of the expected size and translated into a protein. The corresponding open reading frame resulted from the removal of a 57 nt long intron separating two exons of 564 and 510 nt, respectively, and was in agreement with the orientation of the ddB47 cDNA fragment. The presence and precise position of the intron were confirmed by amplifying and sequencing the full length cDNA copy. By aligning the sequences of the full-length genomic copy and the differential display fragment ddB47, the location of the polyadenylation site was mapped at position 64 downstream of the termination codon.

The proposed open reading frame encodes a protein of 357 amino acids. Once the sequence of the genome of two *B. cinerea* strains (B05.10 and T4) was determined (Amselem et al., 2011) it could be concluded that the cloned gene is a single copy gene. In both strains the automatic genome annotation carried out generated the same gene model (BC1G_094092.1 for the B05.10 strain and BofuT4_P148430.1 for the T4 strain) which coincides with the model proposed in this work. In order to experimentally define the position of the genomic DNA sequence being transcribed into at least one of the two mRNAs detected by hybridization we performed a Northern blot analysis using as probes different DNA fragments derived from the 1.960 nt *Sal*I-*Eco*RI genomic DNA fragment cloned (see **Figure 1C**). Three different blots, each containing 20 ug of total RNA extracted from tomato infected leaves collected 120 hpi, one of the moments in which maximal accumulation of the two transcripts was detected (Benito et al., 1996), were probed either with the cDNA fragment ddB47 amplified and labeled by PCR with primers P22 and RT1 (probe I), with a fragment derived from the central part of the proposed coding region amplified and labeled by PCR with primers O47-A and O47-B (probe II) and with a 450 nt *Sal*I-*Kpn*I fragment derived from the region upstream the initiation codon (probe III) and labeled by random priming. Probe I detected again the two mRNAs of 1,4 and 1,0 kb detected originally, while probe II detected only the larger mRNA. With probe III only the 1,4 kb mRNA was detected, but the intensity of the band was very low. This low intensity can be considered an indication of the proximity of the transcription initiation site to the *Kpn*I site. Taking together, these observations indicate that the cDNA fragment ddB47 derives from a *B. cinerea* single copy gene, provisionally named *Bde47*A (from *B*otrytis *d*ifferentially *e*xpressed gene 47A), that encoding the 1,4 kb mRNA, and that there are sequences in the genome with homology to a specific region of that gene, corresponding to the 3′ end of the coding region (very rich in AG residues) and its 3′UTR (amplified as the ddB47 cDNA fragment). These related sequences probably cause the faint bands in the Southern blot analysis. *Bde47*A was soon afterwards renamed *Bcmimp*1 for reasons that will become apparent later.

**FIGURE 2:**
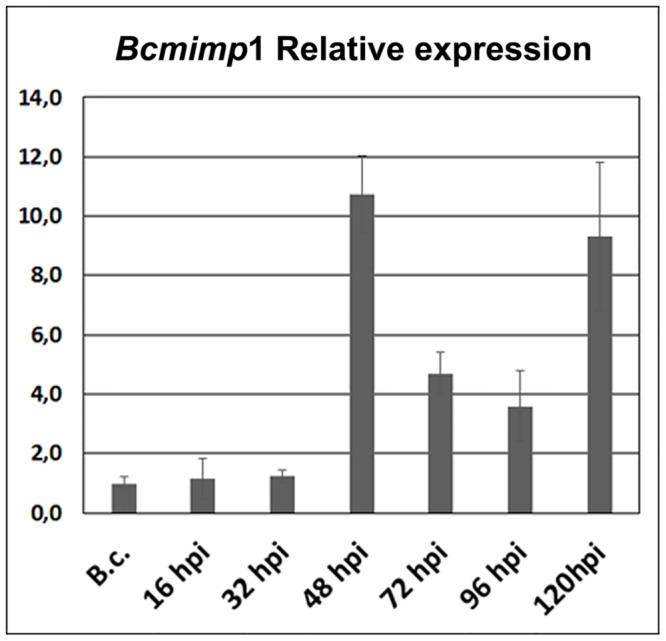
***In planta Bcmimp*1 expression analysis by qPCR.** The level of expression of *Bcmimp*1 at each time point during the interaction is given relative to its level of expression in axenic culture (B.c.- mycelium cultured in liquid B5S/SP medium during 16 h). Each sample is labeled with the time after inoculation (hpi) at which infected tomato leaves were collected.

We performed a new inoculation experiment on detached tomato leaves and samples were collected at the same time points post inoculation: 16, 32, 48, 72, 96, and 120 hpi. Visual inspection of the infected leaves indicated that infection progressed as routinely observed in spray inoculated leaves (Benito et al., 1998), with numerous small primary necrotic lesions appearing until 20–24 hpi during a first infection phase, followed by a quiescent phase between 24 and 48 hpi and finally by the expansion of primary lesions, from 48 to 72 hpi onward, which resulted in full tissue colonization and maceration. With these materials a time-course expression analysis was carried out by qPCR. As shown in **Figure 2**, expression of the *Bcmimp*1 gene displayed a transient expression pattern resembling the pattern observed in northern blot analysis, with peaks of expression at 48 hpi and 120 hpi. Taking together these observations and the results derived from the Southern blot analysis carried out, it can be concluded that *Bcmimp*1 encodes the 1,4 kb mRNA detected previously by differential display and Northern blot analysis and that its expression is enhanced *in planta* following a transient expression pattern.

### The *Bcmimp*1 Protein

The deduced protein sequence encoded by *Bcmimp*1 (357 amino acids) was analyzed using several bioinformatic tools. No significant homology was detected with any previously characterized functional protein domain. InterProScan analysis identified one region, between residues 124 and 283, matching the InterPro motif IPR021836, characteristic of a family of proteins found in bacteria and eukaryotes and functionally uncharacterized, DUF3429. Within this region, four transmembrane domains are predicted in the *Bcmimp*1 protein, located between positions 130 and 152, 178 and 200, 221 and 243, and 263 and 285, respectively. PSORT predicted the protein to be targeted to the mitochondrial IM (probability = 0.900) and MitoFates predicted it possessing a mitochondrial presequence (probability = 0.995) and identified a mitochondrial processing peptidase (MPP) cleavage site at position 22. Therefore, the protein is predicted to be an integral mitochondrial IM protein. As an even number of transmembrane helices are identified, both the N-terminus and the C-terminus of the protein would be orientated towards the same subcellular space, either the mitochondrial matrix or the IS. The C-terminus of the protein is enriched in glutamic acid residues (15 out of the last 32 aminoacids of the protein), conferring an acidic terminal region.

**FIGURE 3:**
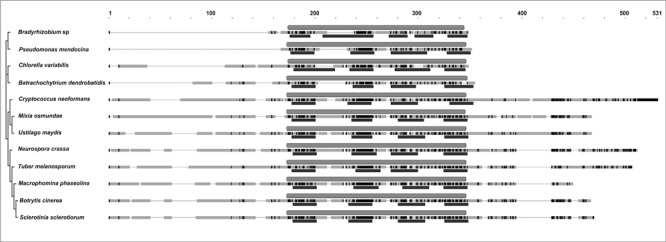
**Alignment and phylogenetic tree of representative selected sequences detected by BLAST analysis with the *Bcmimp*1 encoded protein.** Accession numbers are: YP_005449639 *Bradyrhizobium* sp., YP_001187662 *P. mendocina*, EFN57231.1 *C. variabilis*, EGF79313 *B. dendrobatidis*, XP_774321 *C. neoformans*, GAA97729 *M. osmundae*, XP_761743 *U. maydis*, XP_960098 *N. crassa*, XP_002837349 *T. melanosporum*, EKG13279 *M. phaseolina*, Q0E4W1 *B. cinerea* strain SAS56, XP_001592809 *S. sclerotiorum*. On the right of each species name the sequence is presented as a discontinuous thick line. Thin segments represent gaps introduced to allow optimal alignment between regions represented as thick segments in which similar residues are marked in dark gray. The light gray box above each sequence marks the position occupied by the InterPro motif IPR021836 and the dark gray boxes below correspond to the transmembrane domains predicted in each sequence.

BLAST analysis and database searches identify closely related sequences in members of the fungal kingdom. Orthologs of *Bcmimp*1 appear to be present only in members of the Dikarya, but not in the Chytridiomycota, the Zygomycota and the Glomeromycota. Within the Dikarya, orthologs were identified in the subphyllum Pezizomycotina of the Ascomycota, but not in the subphyllum Saccharomycotina nor in the subphyllum Taphrionomycotina, and in the three subphylla of the Basidiomycota (Agaromycotina, Puccinomycotina, and Ustilaginomycotina). The alignment with the fungal protein sequences shows an overall conservation of protein structure (**Figure 3**). Much more distantly related sequences, both from Bacteria and from other Eukaryota, could be detected by BLAST analysis, but in these cases the similarity was restricted to the regions corresponding to transmembrane domains.

### BCMIMP1 Sub-Cellular Localization

**FIGURE 4:**
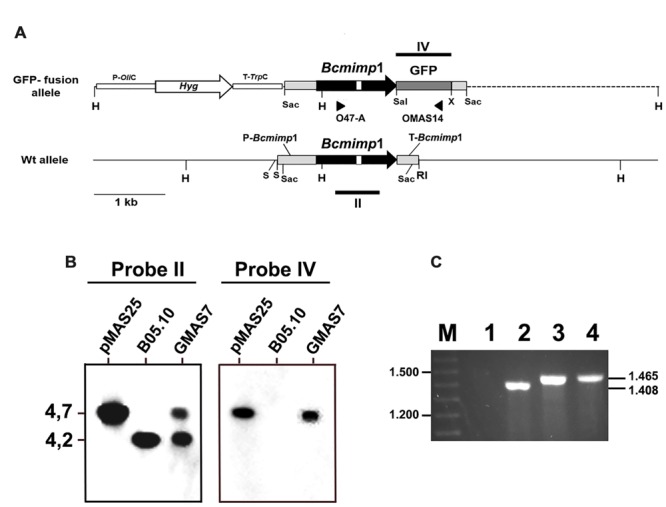
**Molecular characterization of transformant GMAS7. (A)** Restriction map of the *Bcmimp*1 wild type allele and of the *Bcmimp*1-GFP allele cloned in plasmid pMAS25 (this plasmid is represented as a linear molecule but it was used in circular form in transformation experiments). The dotted line represents plasmid pBluescript II SK+ sequences. **(B)** Southern blot analysis of transformant GMAS7. A blot containing 5 μg of genomic DNA from strain B05.10 or from transformant GMAS7, or 2 ng of plasmid pMAS25, digested with *Hind*III and electrophoresed on a 0,7% agarose gel was hybridized with probes derived from the structural region of either the *Bcmimp*1 gene or the GFP gene (Probes II and IV, respectively, in panel **A**). **(C)** Detection of expression of the *Bcmimp*1-GFP fusion allele in transformant GMAS7. cDNA from the wild type strain B05.10 (lane 1) or from transformant GMAS7 (lane 2) mycelium (cultured during 16 h in B5S/SP medium), genomic DNA from transformant GMAS 7 (lane 3), and plasmid pMAS25 (lane 4) were used as templates in PCR amplifications carried out with primers O47-A and OMAS14. PCR products were resolved on a 0.7% agarose gel. Differences is size between the GMAS7 cDNA and genomic DNA derived PCR products are due to the presence of an intron in the *Bde47*A gene. M: 100 bp ladder size marker. RI, *Eco*RI; H, *Hind*III; S, *Sal*I; Sac, *Sac*I; X, *Xba*I.

In order to study the subcellular localization of the protein encoded by the *Bcmimp*1 gene, a GFP fusion was generated. To this end the coding region of the GFP gene was fused in frame to the 3′-end of the *Bcmimp*1 gene and cloned in vector pMAS25 (which also includes a hygromycin resistance expression cassette) (**Figure 4A**). In this way, the upstream and downstream expression regulatory signals of *Bcmimp*1 would be maintained unaltered in the fusion construct. Protoplasts of *B. cinerea* were transformed with pMAS25 and several transformants were obtained. Transformant GMAS7 was selected as a representative transformant of a single integration event in which the resident wild type allele remains unaltered (**Figure 4B**). Expression of the fusion allele in transformant GMAS7 during saprophytic growth was demonstrated by RT-PCR. As shown in **Figure 4C**, using a primer combination which includes a *Bcmimp*1 specific primer (O47A) and a GFP specific primer (OMAS14) (**Figure 4A**) a PCR fragment of the expected size (1.408 nt) was amplified from cDNA derived from GMAS7 mycelium, but not from B05.10 mycelium. A slightly larger fragment, 1.465 nt is size, derived from the genomic copy of the fusion allele was amplified from genomic DNA of transformant GMAS7.

**FIGURE 5:**
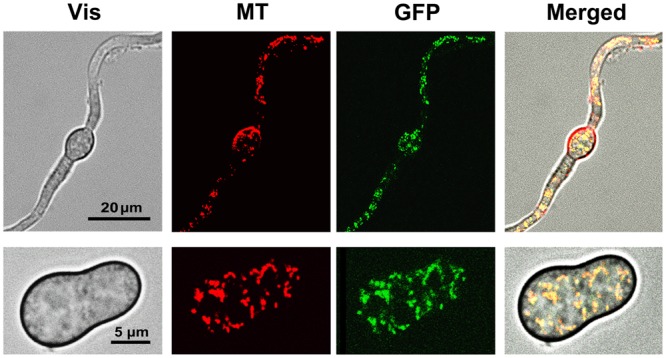
**Subcellular localization of the BCMIMP1-GFP fusion protein.** Mycelium (cultured during 16 h, upper row images) or germinating spores (cultured during 5 h, lower row images) from transformant GMAS7 grown in B5S/SP medium were loaded with Mitotracker Red dye and analyzed on a confocal laser microscope. Images were taken using the conditions and filters sets for Red (MT) or Green (GFP) fluorescence detection. (Vis): Light transmission images. (Merged): Overlapped MT, GFP, and Vis images.

Determination of the sub-cellular localization of the GFP tagged protein was performed by confocal laser microscopy. Germinating spores and mycelium (incubated during 5 and 16 h, respectively, in B5S/SP medium) of transformant GMAS7 were loaded with the mitochondria-specific dye MitoTracker Red. Samples were then analyzed under the microscope and images were captured under conditions allowing the detection of either the red fluorescence of MitoTracker Red or the green fluorescence of GFP. As shown in **Figure 5**, the red and green fluorescence dotted patterns fully overlapped both in young branching mycelium samples and in germinating spores samples, indicating colocalization of the *Bcmimp*1-GFP fusion protein and the mitochondria. The same dotted pattern was observed with several transformants obtained with plasmid pMAS25 (not shown). These observations provide experimental confirmation that the protein encoded by gene *Bcmimp*1 is targeted to the mitochondria. Once the subcellular localization of the encoded protein was confirmed the name *Bcmimp*1, for *Botrytis cinerea* gene for mitochondrial inner membrane protein 1, was definitively adopted (Accession No. AM400897).

### Functional Characterization of *Bcmimp*1

**FIGURE 6:**
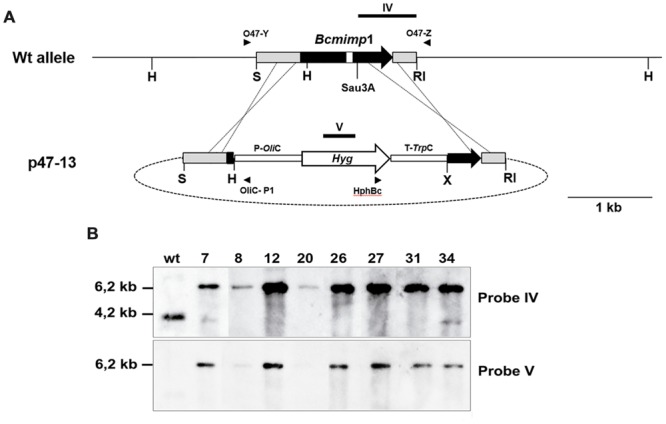
**Identification of *ΔBcmimp*1 mutants. (A)** Restriction map of the genomic copy of gene *Bcmimp*1 and of the gene replacement vector p47-13. In this vector the central part of the structural region of *Bcmimp*1 has been replaced by a hygromycin resistance cassette. The annealing positions of the primers used for PCR detection of homologous recombination events at either flanking region are indicated. The target sites of the probes used for hybridization (probes IV and V) are also indicated. **(B)** Southern blot analysis of selected transformants obtained with plasmid p47-13. A blot containing 5 μg of *Hind*III digested genomic DNA from the *B. cinerea* wild type strain B05.10 or from the candidate transformants identified by PCR, was successively hybridized with a probe derived from the 3′ end of the *Bcmimp*1 structural region and its 3′UTR cloned in plasmid p47-13 (probe IV) of from the hygromycin resistance gene (probe V). RI, *Eco*RI; H, *Hind*III; K: S, *Sal*I; Sau; *Sau*3A; X: *Xba*I.

In order to examine the role of *Bcmimp*1 in *B. cinerea*, targeted deletion mutants were generated by applying a gene replacement strategy based on the use of plasmid p47-13 to transform protoplasts of *B. cinerea* strain B05.10 (see **Figure 6A**). 8 out of 50 transformants obtained with plasmid p47-13 were shown by PCR to carry the two diagnostic bands expected in case of replacement of the *Bcmimp*1 wild type allele. These candidates were characterized by Southern blot analysis. As shown in **Figure 6B**, transformants 8, 12, 20, 26, 27, and 31 were true mutant strains as the single 4,2 kb *Hind*III band detected by hybridization with probe IV representing the wild type allele was substituted by the expected 6,2 kb *Hind*III band (also detected with probe V, derived from the hygromycin cassette). No additional copies of the hygromycin resistance cassette integrated elsewhere were detected in these transformants. In transformants 7 and 34, the wild type allele was detected in addition to the replacement allele, indicating that these transformants are still heterokaryotic. For functional characterization, transformants 12, 26, and 27 were compared with two control strains: transformant pOHT-4, obtained with an empty vector plasmid pOHT, and strain B05.10-C4, derived from a single regenerated protoplast cultured in the absence of hygromycin selection.

**FIGURE 7:**
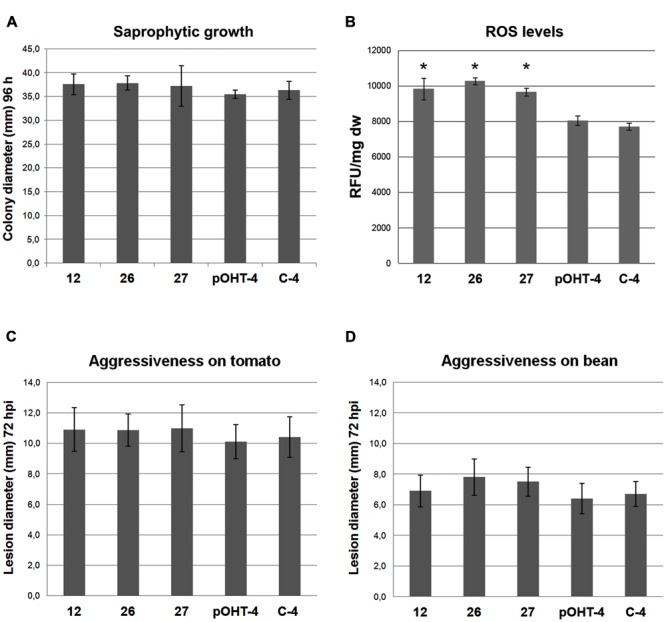
**Phenotypic characterization of the *ΔBcmimp*1 mutant strains 12, 26, and 27, in comparison with two control strains, pOHT-4 and C4. (A)** Saprophytic growth capacity was estimated as the colony diameter (mm) in B5S/SP medium plates after 96 h. **(B)** Estimation of the levels of total reactive oxygen species (ROS) produced by the strains analyzed. For each strain fluorescence was measured in 200 μl volumes containing the cells collected from 1 ml of B5S/SP medium spore suspensions cultured during 5 h once loaded with H_2_DCFDA. Fluorescence is expressed as arbitrary Fluorescence Units per mg of dry weight (FU/mg dw). **(C,D)** Evaluation of the aggressiveness of the strains analyzed on tomato **(C)** and on bean **(D)** leaves. Aggressiveness was estimated by measuring the lesion diameter generated 72 hpi from 5 μl drops containing 500 spores of the different strains in B5S/SP medium. In each panel the data presented are the average values of three experiments. **(B)**, (^∗^) indicate significant differences (*p* < 0.05) between the mutant strain and the reference strains, which grouped together.

The three *ΔBcmimp*1 transformants did not show any obvious morphological and/or developmental alteration in comparison with the two reference strains and their sporulation capacity and germination efficiency was not affected (not shown). Their capacity to grow in synthetic media was evaluated in a minimal medium, B5S/SP. ANOVA analysis of radial growth showed no significant difference in their capacity to grow on this medium when compared to the reference strains (**Figure 7A**).

As the BCMIMP1 protein is targeted to the mitochondria and mitochondria constitute a major source of endogenously produced ROS in eukaryotic cells, we evaluated differences in the amount of ROS produced by the *ΔBcmimp*1 mutants in comparison with the reference strains. To this end, conidia suspensions of the selected stains were incubated during 5 h in liquid B5SP. The germlings were stained with the fluorescent dye 2′,7′-dichlorodihydrofluorescein diacetate (H_2_DCFDA) and fluorescence was quantified. The results shown in **Figure 7B** indicate that the three *ΔBcmimp*1 mutant strains produce higher levels of ROS than the pOHT-4 and the C4 reference strains. Statistical analysis of the data demonstrated that the observed differences are significant (*p* < 0.05).

To determine if deletion of *Bcmimp*1 affects pathogenicity, the ability of the mutants to infect two different hosts, tomato and bean, was evaluated in infection assays carried out on intact plants under controlled inoculation conditions. On tomato, the average lesion diameters generated by the three mutants was very similar to one another, and consistently slightly higher that the lesion diameter generated by the two reference strains (**Figure 7C**). However ANOVA analysis demonstrated that the observed differences are not significant. A similar situation was found in bean (**Figure 7D**). From these observations it can be concluded that *Bcmimp*1 does not play an essential role in pathogenicity in *B. cinerea* under the experimental conditions considered in this work.

## Discussion

Mitochondria are eukaryotic organelles with a decisive role in many cellular processes, like energy metabolism and programmed cell death. The IM hosts respiratory chain proteins organized in supramolecular complexes (Boekema and Braun, 2007; Chaban et al., 2014) and a large number of transport proteins and ion channels, responsible for the mitochondrial role in cell homeostasis. Many of these proteins are considered essential, as the shortage or fluctuation of their activity lead to the production of high levels of ROS, related to pathological and aging processes (Joseph-Horne et al., 2001). In humans, a large number of mitocondrial disorders have been found to be biochemically characterized by defective oxidative phosphorylation, being the consequence of mutations either in mitochondrial or in nuclear genes encoding subunits of the respiratory chain complexes or in nuclear genes encoding proteins that are essential for the proper import, maturation and assembly of the complexes (Calvo and Mootha, 2010). We cloned *Bcmimp*1, a *B. cinerea* nuclear gene encoding a protein targeted to the mitochondria. Colocalization studies of a BCMIMP1-GFP fusion protein using confocal laser microscopy, unequivocally demonstrated that BCMIMP1 is a mitochondrial protein. Although it does not allow to determine its precise location within the mitochondria, the bioinformatic analysis performed strongly support its localization at the IM. As the protein does not contain any known functional domain (despite extensive database searches), but it contains several transmembrane helixes regularly spaced in the central region of the protein, BCMIMP1 can be proposed to be a mitochondrial IM integral protein. The central region matches the InterPro motif IPR021836, characteristic of the protein family DUF3429, including proteins found in bacteria and eukaryotes. We cannot infer any functional role for BCMIMP1 since no member of this family has been functionally characterized previously. This paper provides the first report on the functional characterization of a member of this family. *Bcmimp*1 has orthologs only within the fungal kingdom, but these orthologs are not present in all the fungal taxa, showing up only in some Ascomycetes and in the Basidiomycetes. The low homology with proteins from bacteria and algae are likely more a consequence of the structural similarity derived from the conservation of the transmembrane domains organization, rather than from any functional relationship.

The *Bcmimp*1 gene was identified on the basis of its differential expression *in planta*. The fact that two different mRNAs were detected on a Northern blot with a single cDNA derived probe initially introduced some elements of ambiguity and complexity. The Southern and Northern analysis carried out, together with the alignments of the cDNA (both the ddB47 fragment and the CDS derived copies) and genomic sequences, explain the observations generated and demonstrate that the gene cloned, *Bcmimp*1, is the gene encoding the 1,4 kb mRNA detected in the original differential display analysis. The AG-rich regions in the cDNA probe used during the initial hybridization experiments caused weak hybridization to additional regions of the *B. cinerea* genome and to a mRNA of 1,0 kb.

Enhanced expression is considered an indication of the participation of a gene product in a given process. We applied this strategy in an attempt to identify *B. cinerea* genes essential for pathogenicity and *Bcmimp*1 is one of such genes. Pathogenicity tests carried out with three independent *ΔBcmimp*1 mutants and two control strains demonstrate that the BCMIMP1 protein is not a pathogenicity factor, as the mutants did not show reduced aggressiveness in either of the two hosts investigated. The transient expression pattern of the gene *in planta* probably reflects the situation the fungus is experiencing during the establishment and progress of the infection. Enhanced expression coincided with the time points at which primary lesions start expansion (48 hpi) and when maceration of the plant tissues occurs (120 hpi). As these are the moments of most profuse fungal growth, it can be reasoned that these should also be the most energy demanding stages for the fungus during the infection cycle. Since energy production is the main function of mitochondria, the up-regulation of genes encoding mitochondrial components during certain infection stages might reflect the necessity of mitochondrial biogenesis, or of activation of mitochondrial energy metabolism in order to satisfy the energy demands of the fungus.

Although the *ΔBcmimp*1 mutants do not show an obvious morphological or developmental alteration during saprophytic growth, they produce higher levels of ROS. It is known that mitochondria are the main source of ROS in most cell types, where they are formed as a byproduct of respiration upon incomplete reduction of oxygen at several sites of the electron transport chain (Davies, 1995; Turrens, 2003). In most cases, alterations in components of the electron transport chain result in increased ROS production (Zuin et al., 2008). On the basis of these considerations it can be proposed that the higher levels of ROS produced by *ΔBcmimp*1 mutants are possibly a consequence of BCMIMP1 depletion from the mitochondrial IM, which somehow compromises the normal functioning of the mitochondria. At this moment, no conclusion can be drawn about the nature of the physiological alteration resulting from the absence of the BCMIMP1 protein, which appears to be an integral mitochondrial IM protein with the amino- and the carboxy-terminus oriented towards the same cellular space, either the mitochondrial matrix or the IS, and has a carboxy-terminus highly acidic. It has been demonstrated that an acidic carboxy-terminus in some proteins can play an essential role in protein-protein interactions (Ohkuma et al., 1995; Kong and Richardson, 1998). Possibly, BCMIMP1 is an integral mitochondrial IM protein required for the correct organization of the IM or of its components and its carboxy-terminus plays an important role in the establishment of protein interactions essential for the correct function of the protein complexes anchored into it.

In the functional analysis performed *in planta* we did not find a reduction in virulence in the *ΔBcmimp*1 mutants in either host. On the contrary, although the differences found were not statistically significant, we consistently observed a slightly larger average lesion size in comparison with the wild type and control strains. This small increase in virulence can be a side effect of the high levels of ROS produced by the mutant strains. Evidence accumulates indicating that necrotrophic fungi need and even stimulate the plant oxidative burst response and that they can produce ROS and contribute to the oxidative status *in planta* (reviewed by Heller and Tudzynski, 2011). Mitochondria are considered an internal source of ROS which can contribute to ROS levels in the interaction zone. In such a scenario the higher levels of ROS produced by the *ΔBcmimp*1 mutants could explain the minor differences in lesion size consistently observed for the three mutants on the two hosts considered.

This work demonstrates that *Bcmimp*1 is not a pathogenicity factor but the molecular and functional analysis carried out provides useful information to gain insight into the physiological status of the pathogen during the different stages of the infection process. As more genes are characterized the knowledge of the infection process will be more detailed and complete.

## Author Contributions

Dr. EB is the corresponding author. He is the researcher responsible of the conceptual and experimental design, supervision of work, data adquisition, analysis and interpretation, and preparation of the manuscript. He asumes full responsability on the accuracy or integrity of work being presented. Drs. DB-P, DS, MA, and JD-M performed a substantial part of the experimental research, generated and analyzed data and participated in the drafting of the manuscript. Drs. AE and JvK made substantial contribution to the conception of the work and to the analysis and interpretation of data and to the writing of the manuscript. They all participated in the revision and final approval of the version to be published and are in agreement to be accountable for all aspects of the work.

## Conflict of Interest Statement

The authors declare that the research was conducted in the absence of any commercial or financial relationships that could be construed as a potential conflict of interest.
